# Non-Ischemic Cerebral Energy Dysfunction at the Early Brain Injury Phase following Aneurysmal Subarachnoid Hemorrhage

**DOI:** 10.3389/fneur.2017.00325

**Published:** 2017-07-10

**Authors:** Laurent Carteron, Camille Patet, Daria Solari, Mahmoud Messerer, Roy T. Daniel, Philippe Eckert, Reto Meuli, Mauro Oddo

**Affiliations:** ^1^Department of Intensive Care Medicine, CHUV-University Hospital, University of Lausanne, Lausanne, Switzerland; ^2^Neuroscience Critical Care Research Group, CHUV-University Hospital, University of Lausanne, Lausanne, Switzerland; ^3^Department of Neurosurgery, CHUV-University Hospital, University of Lausanne, Lausanne, Switzerland; ^4^Department of Radiology, CHUV-University Hospital, University of Lausanne, Lausanne, Switzerland

**Keywords:** cerebral microdialysis, subarachnoid hemorrhage, neuroenergetics, hyperemia, early brain injury

## Abstract

**Background:**

The pathophysiology of early brain injury following aneurysmal subarachnoid hemorrhage (SAH) is still not completely understood.

**Objective:**

Using brain perfusion CT (PCT) and cerebral microdialysis (CMD), we examined whether non-ischemic cerebral energy dysfunction may be a pathogenic determinant of EBI.

**Methods:**

A total of 21 PCTs were performed (a median of 41 h from ictus onset) among a cohort of 18 comatose mechanically ventilated SAH patients (mean age 58 years, median admission WFNS score 4) who underwent CMD and brain tissue PO_2_ (PbtO_2_) monitoring. Cerebral energy dysfunction was defined as CMD episodes with lactate/pyruvate ratio (LPR) >40 and/or lactate >4 mmol/L. PCT-derived global CBF was categorized as oligemic (CBF < 28 mL/100 g/min), normal (CBF 28–65 mL/100 g/min), or hyperemic (CBF 69–85 mL/100 g/min), and was matched to CMD/PbtO_2_ data.

**Results:**

Global CBF (57 ± 14 mL/100 g/min) and PbtO_2_ (25 ± 9 mm Hg) were within normal ranges. Episodes with cerebral energy dysfunction (*n* = 103 h of CMD samples, average duration 7.4 h) were frequent (66% of CMD samples) and were associated with normal or hyperemic CBF. CMD abnormalities were more pronounced in conditions of hyperemic vs. normal CBF (LPR 54 ± 12 vs. 42 ± 7, glycerol 157 ± 76 vs. 95 ± 41 µmol/L; both *p* < 0.01). Elevated brain LPR correlated with higher CBF (*r* = 0.47, *p* < 0.0001).

**Conclusion:**

Cerebral energy dysfunction is frequent at the early phase following poor-grade SAH and is associated with normal or hyperemic brain perfusion. Our data support the notion that mechanisms alternative to ischemia/hypoxia are implicated in the pathogenesis of early brain injury after SAH.

## Introduction

Increasing evidence suggests that early events occurring during the first 72 h following ictus onset, before the occurrence of secondary delayed neurological deterioration, substantially contribute to the pathophysiology and outcome of aneurysmal subarachnoid hemorrhage (SAH). This so-called early brain injury phase includes multiple physiological derangements that exacerbates the primary cerebral insult and may eventually worsen patient prognosis (1–6). While research efforts have predominantly focused on cerebral hemodynamics and perfusion, and the comprehension of mechanisms involved in brain ischemia, it is now increasingly recognized that a number of alternative “non-ischemic” mechanisms are implicated in the pathogenesis of early brain injury (7).

Energy dysfunction has emerged as one potential pathogenic determinant of cerebral damage following acute brain injury. Using a combination of intra-cerebral microdialysis (CMD)—to quantify regional brain metabolism—and neuroimaging techniques, to assess brain perfusion and oxygenation, several studies have reported profound derangements in the regulation of neuroenergetics in the absence of ischemia at the early phase of acute brain injury (8–15). So far, however, cerebral energy dysfunction has mainly been described following neurotrauma (16–19). Whether cerebral energy dysfunction contributes to early brain injury following SAH has not been extensively studied; moreover, its exact nature and potential cerebral blood flow (CBF) correlates have not been precisely characterized.

The aim of this study in comatose SAH patients who underwent advanced multimodal neuromonitoring was to examine the relationship of cerebral energy metabolism (using CMD) with brain oxygenation (using brain tissue PO_2_, PbtO_2_) and CBF. CBF was quantified using brain perfusion CT (PCT) performed at the early phase of SAH. We hypothesized that early brain injury following SAH leads to an increase in neurochemical markers of cerebral energy dysfunction [CMD lactate and lactate/pyruvate ratio (LPR)] and brain cell damage (CMD glycerol) despite the absence of cerebral ischemia/hypoxia.

## Materials and Methods

### Patients

Subjects were part of a retrospective observational cohort of patients with SAH admitted to the Intensive Care Unit who underwent CMD/PbtO_2_ monitoring and brain PCT as part of standard care. Indication for intracranial monitoring was a post-resuscitation Glasgow Coma Scale <9. Subjects were recruited over a 2-year period (March 2013–April 2015); only patients who had complete CMD/PbtO_2_ data and in whom brain PCT was performed within 96 h from SAH were included in the present study. A subset of the present cohort was also included in another clinical investigation that evaluated that value of CMD to detect delayed cerebral ischemia, submitted elsewhere. Approval for the study was obtained from our local Ethical Committee, and a waiver of consent was given due to the retrospective nature of the study.

### Cerebral Microdialysis Monitoring

Cerebral microdialysis consisted of a CMA 70 catheter (20 kDa cutoff, CMA Microdialysis AB^®^, Stockholm, Sweden; flow rate 0.3 µL/min), inserted through a triple-lumen bolt into subcortical white matter generally in the non-dominant frontal lobe in an area situated between the anterior and the middle cerebral artery territories. CMD samples were collected hourly and analyzed immediately at the bedside with the ISCUS flex system (CMA Microdialysis AB^®^) for brain extracellular concentrations of lactate, pyruvate, glucose, glutamate, and glycerol. First hour of monitored data, artifacts, and values outside obvious physiological ranges were manually excluded. In all patients, PbtO_2_ (Licox^®^, Integra Neurosciences, Plainsboro, NJ, USA) and intracranial pressure (ICP; Codman^®^, Raynham, MA, USA) probes were also inserted, adjacent to the CMD catheter. The correct placement of all probes was verified within 24 h by a follow-up non-contrast head CT scan.

### General Patient Management

Patients were managed according to a written standardized algorithm, in line with international guidelines (20). After initial stabilization, the ruptured aneurysm was secured either by surgical clipping or by endovascular coiling. All patients were mechanically ventilated, targeting a P_a_O_2_ of 90–100 mm Hg and a P_a_CO_2_ of 35–40 mm Hg. Cerebral perfusion pressure (CPP) was maintained at least >70 mm Hg, if needed with the use of fluids (crystalloids) and vasopressors (norepinephrine). Blood glucose was maintained at 6–8 mmol/L, and enteral nutrition was started within 48 h.

### Brain Perfusion Computed Tomography

Neuroimaging was conducted in stable conditions of ICP, and systemic hemodynamics. PCT was performed using a multi-detector row CT Lightspeed (GE medical systems, Milwaukee, WI, USA). Scanning was initiated 5 s after injection of 50 mL of iohexol (300 mg/mL of iodine; GE Healthcare Europe), perfused at a rate of 5 mL/s, with the following parameters: 80 kV, 240 mAs, 0.4 rotations/s, and total duration of 50 s. The series evaluated 16 adjacent 5-mm-thick sections of brain parenchyma. Postprocessing of PCT data was performed by an experienced neuroradiologist, masked to CMD and PbtO_2_ variables, using a dedicated software (Brilliance Workspace Portal^®^, Philips Medical Systems, Cleveland, OH, USA), which uses the central volume principle using deconvolution to measure the mean transit time (MTT). Cerebral blood volume (CBV) is calculated from the time-enhancement curves, and CBF is derived from the equation: CBF = CBV/MTT.

We defined four main regions of interest (ROIs): right and left middle cerebral artery, right and left anterior cerebral artery. CBF from posterior and cerebellar arteries territories were not analyzed in this study. Three-dimensional reconstruction was processed with Carestream Vue PACS (Carestream Health, Rochester, NY, USA) using a series of the thin-slice enhanced brain CT. Because intracranial probes were located in the white matter, ROIs were drawn in areas of predominant white matter to allow concordant measurement of global supratentorial perfusion in the same type of tissue. All procedures and the ROIs’ characteristics were selected in line with previous studies (16). Global CBF was defined as the average of 4 ROIs’ CBF. Once the postprocessing completed, each PCT was then categorized as *ischemic* (global CBF < 28 mL/100 g/min), *normal* (global CBF 28–66 mL/100 g/min), or *hyperemic* (global CBF > 66 mL/100 g/min) (21, 22).

### Determination of Episodes with Cerebral Energy Dysfunction

Episodes with cerebral energy dysfunction were defined by LPR >40 and/or lactate >4 mmol/L in brain extracellular fluid, according to recent consensus guidelines (23). Simultaneous levels of CMD glutamate, glycerol, as well as PbtO_2_ and ICP (averaged to the hour previous to CMD sampling), were also collected. For the purpose of the study, we analyzed CMD data closest to the time of PCT data. Identification of episodes of energy dysfunction was conducted by LC and MO, blinded to PCT data.

### Statistical Analysis

Variables of interest included cerebral metabolic (CMD derived) data, PbtO_2_, and PCT findings. Statistical analysis was performed using JMP-10^®^ package software (SAS Institute, Cary, NC, USA). Results were expressed as mean ± SD or median and interquartile range, and comparisons between groups were explored using Student’s *t*-test or Mann–Whitney test as appropriate. Linear correlations were tested using the non-parametric Spearman’s correlation coefficient. Significance was set at a *p* < 0.05.

## Results

### Patient Characteristics

Out of a total of 29 patients who underwent CMD monitoring during the study period, complete dataset was available for 18 non-consecutive comatose subjects who underwent 21 PCTs during the early post-injury phase [a median of 41 (25–73) h from ictus onset]. Patients were predominantly women (16/18), mean age was 58 ± 9 years, and modified Fisher scale was 3 (1 patient) or 4 (17 patients). All patients were sedated and mechanically ventilated at the time of CT perfusion.

### Brain PCT during the Early Brain Injury Phase after SAH

Brain PCT data are summarized in Table 1. No significant differences for CBF were found between the different vascular territories. Global CBF averaged 57 ± 14 mL/100 g/min: among the 21 PCTs, 14 showed normal CBF while 7 were classified as hyperemic. Importantly, among the three patients in whom brain PCT was performed twice, CBF findings were concordant over the two repeated measurements (i.e., two patients with normal CBF, and one patient with hyperemic CBF). When examining concomitant cerebral physiologic variables (*n* = 157 h of intracranial monitoring, Table 2), we found that ICP and PbtO_2_ were on average within normal ranges. In contrast, brain extracellular concentrations of LPR, glutamate and glycerol were all elevated (Table 2), indicating substantial cerebral metabolic abnormalities in the early brain injury phase following SAH in our patient cohort, despite the absence of cerebral ischemia/hypoxia.

**Table 1:** Cerebral blood flow (CBF) during the early brain injury phase following aneurysmal subarachnoid hemorrhage.

Region of interest	CBF, mL/100 g/min
Right middle cerebral artery	59.6 (29.3–96.8)
Left middle cerebral artery	63.9 (29.4–87.7)
Right anterior cerebral artery	55.9 (28.1–97.8)
Left anterior cerebral artery	62.2 (26.5–83.7)
Global	59.7 (27.7–84.5)

*CBF was calculated with brain perfusion CT (*n* = 21, performed a median of 41 h from ictus onset) in four different regions of interest (ROIs); global CBF was defined as the average of CBF from the four different ROIs*.

*Data are expressed as mean (range)*.

**Table 2:** Global cerebral blood flow (CBF), main brain physiologic, and cerebral metabolic variables at the time of brain perfusion CT.

Variable	Value
**Cerebral perfusion**	
CBF, mL/100 g/min	57 ± 14
**Cerebral hemodynamics**	
Brain tissue PO_2_, mm Hg	25 ± 9
Intracranial pressure, mm Hg	13 ± 7
Cerebral perfusion pressure, mm Hg	77 ± 9
**Cerebral metabolism**	
Brain extracellular lactate/pyruvate ratio	42 ± 16
Brain extracellular lactate, mmol/L	5.3 ± 3.3
Brain extracellular pyruvate, μmol/L	141 ± 99.2
Brain extracellular glycerol, μmol/L	121 ± 65
Brain extracellular glutamate, μmol/L	29 ± 39
Brain extracellular glucose, mmol/L	1.1 ± 0.7

*Data are presented as mean ± SD. Brain extracellular concentrations were measured with cerebral microdialysis*.

### Cerebral Energy Dysfunction during the Early Brain Injury Phase following SAH

**Figure 1:**
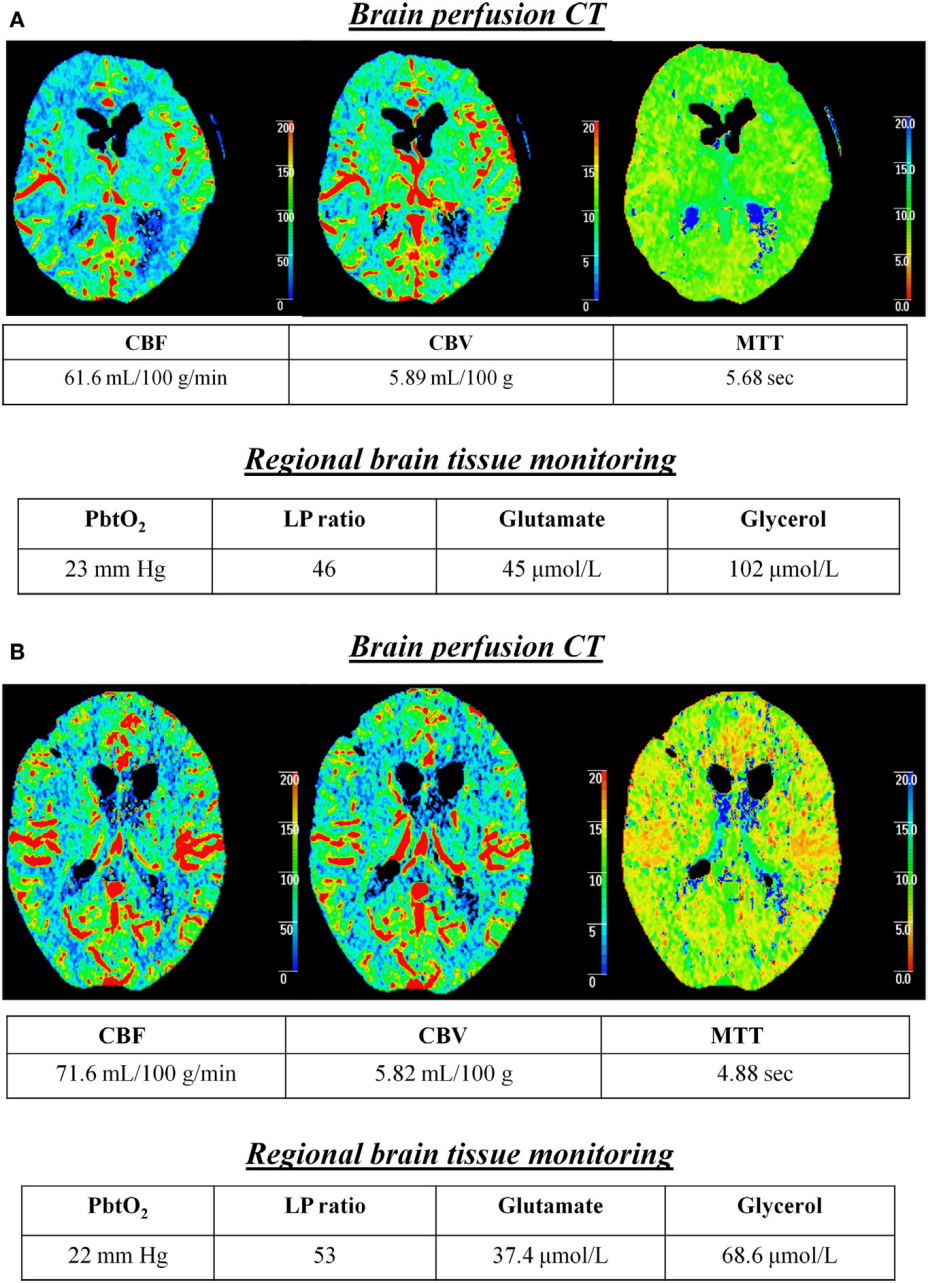
Cerebral energy dysfunction is associated with normal or hyperemic CBF in the early brain injury phase of aneurysmal subarachnoid hemorrhage. Illustrative examples of two patients in whom cerebral energy dysfunction was associated with brain perfusion CT (PCT) showing normal **(A)** or hyperemic **(B)** CBF. Time from PCT to subarachnoid hemorrhage was 16 h in patient **(A)** and 38 h in patient **(B)**. Brain extracellular concentrations of lactate/pyruvate (LP) ratio, glutamate, and glycerol were measured with a 20 kDa cerebral microdialysis catheter that was located in the subcortical white matter, adjacent to a brain tissue PO_2_ (PbtO_2_) probe. Abbreviations: CBF, cerebral blood flow; CBV, cerebral blood volume; MTT, mean transit time.

The relationship of cerebral energy dysfunction (*n* = 103 h of CMD samples, average duration 7.4 h) with global CBF was further analyzed, according to CBF classification (normal vs. hyperemic). Figure 1 illustrates two representative patients in whom cerebral energy dysfunction was associated with normal (Figure 1A) or hyperemic (Figure 1B) CBF on PCT, and absence of low PbtO_2_.

Complete CMD data were available for 19 PCTs: episodes of cerebral energy dysfunction were observed in 14/19 PCT measurements, corresponding to 66% of total CMD samples examined. Nine PCTs showed normal CBF while five were hyperemic. Cerebral metabolic abnormalities were more pronounced in hyperemic than normal CBF (Table 3): cerebral hyperemia was associated with higher levels of brain extracellular LPR (54.1 ± 11.9 vs. 42.1 ± 6.5 when CBF was normal, *p* < 0.0001) and glycerol (157.0 ± 76.1 vs. 95.1 ± 41.4 µmol/L, *p* < 0.0001). Brain CMD glutamate also was higher when CBF was hyperemic (38.0 ± 51.5 vs. 25.9 ± 23.5 µmol/L when CBF was normal), although this did not reach statistical significance (*p* = 0.18). As shown in Table 3, ICP and PbtO_2_ did not differ between the two CBF conditions: only a minority of samples (16%) had elevated ICP >20 mm Hg or PbtO_2_ <15 mm Hg (11%).

**Table 3:** Episodes of cerebral energy dysfunction categorized according to cerebral blood flow (CBF), dichotomized as normal vs. hyperemic.

	Normal CBF (*n* = 9)	Hyperemic CBF (*n* = 5)	*p*-Value
CBF, mL/100 g/min	44.6 ± 13.3	69.9 ± 4.9	<0.0001
Brain lactate/pyruvate ratio	42 ± 7	54 ± 12	<0.0001
Brain glycerol, μmol/L	95.1 ± 41.4	157.0 ± 76.1	<0.0001
Brain glutamate, μmol/L	25.9 ± 23.5	38.0 ± 51.5	0.18
Brain tissue PO_2_, mm Hg	22.8 ± 12.2	26.2 ± 6.3	0.18
Intracranial pressure, mm Hg	14 ± 9	14 ± 6	0.75

**Figure 2:**
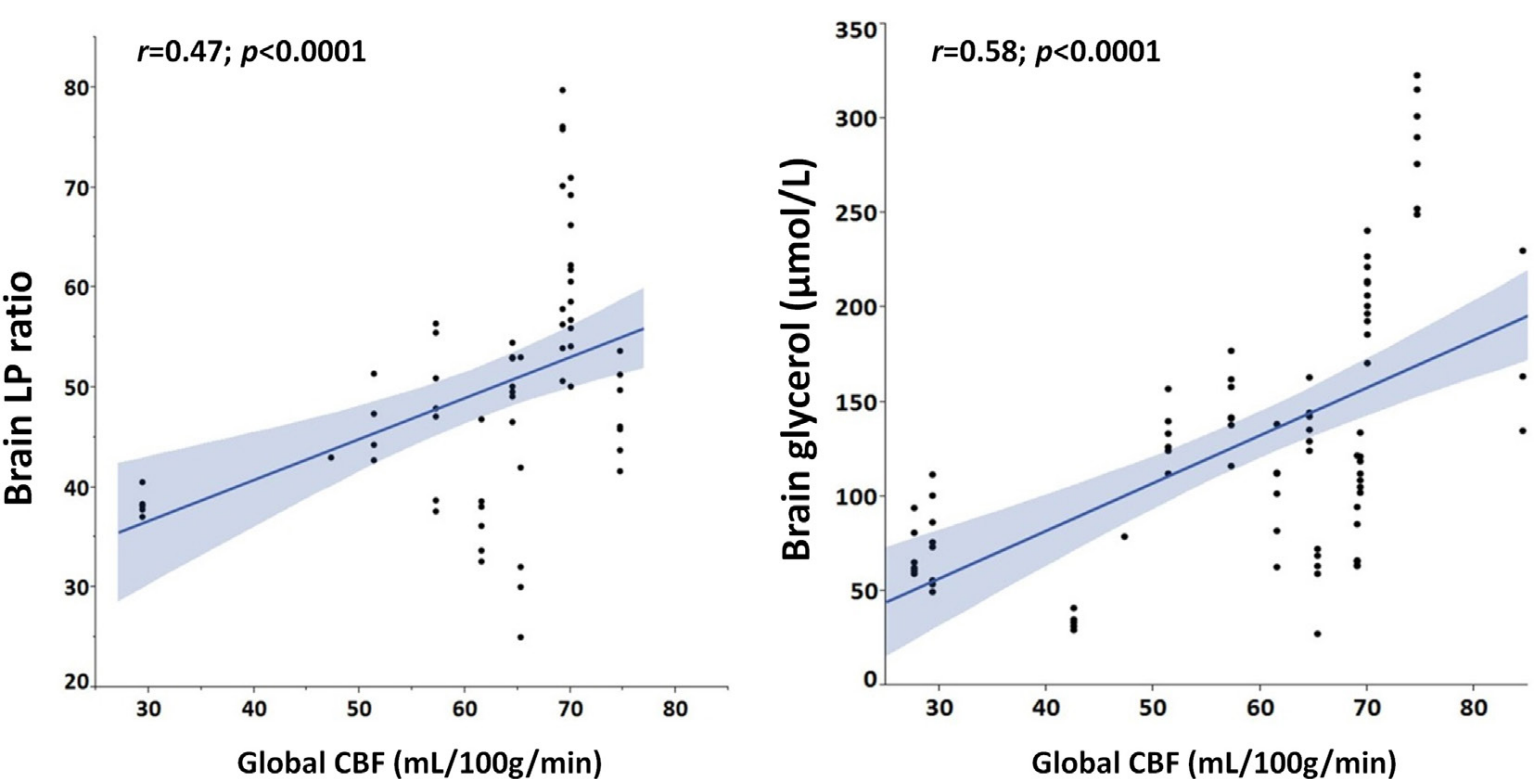
Hyperemic cerebral blood flow (CBF) is positively correlated with cerebral metabolic and cellular distress. Graphs illustrate Spearman’s linear correlations of global CBF, measured with perfusion CT, with brain extracellular concentrations of lactate/pyruvate (LP) ratio and glycerol.

As shown in Figure 2, Spearman’s linear correlation coefficient analysis found a positive linear correlation of higher global CBF with both elevated brain LPR (*r* = 0.47, *p* < 0.0001) and brain glycerol (*r* = 0.58, *p* < 0.0001) during episodes of cerebral energy dysfunction, thereby suggesting that cerebral hyperemia correlated with more pronounced derangements in brain interstitial tissue biochemistry.

Global CBF was higher during episodes with cerebral energy dysfunction (*n* = 14; CBF 59.2 ± 15.7 mL/100 g/min) vs. those without (*n* = 5; CBF 51.2 ± 7.9 mL/100 g/min, *p* = 0.01).

## Discussion

Our data combining regional brain tissue monitoring with perfusion head CT scan demonstrate that cerebral energy dysfunction may occur despite absence of brain hypoxia/ischemia, since PbtO_2_ was on average within normal ranges and cerebral perfusion was normal, or even supranormal. This clinical study identifies cerebral energy dysfunction as a novel pathogenic determinant of early brain injury following SAH. Our findings further suggest that, at the early phase of SAH, interventions targeted to cerebral perfusion and oxygenation may not be sufficient to prevent further secondary brain damage. It is plausible to conceive that alternative strategies aimed at improving cerebral energy metabolism may be of value in this setting.

### Cerebral Perfusion in the Early Brain Injury Phase of SAH

All PCTs were showing normal to supranormal CBF. Our findings (see Table 1) are in line with previous clinical observations showing that CBF measured at the early acute phase following SAH was on average above 54 mL/100 g/min, irrespective of later occurrence of delayed cerebral ischemia (24, 25). Normal CBF was associated with normal PbtO_2_ and ICP values. Thus, our study combining cerebral perfusion imaging with monitoring of regional brain physiology did not reveal evidence of cerebral ischemia or hypoxia in our patient cohort, at least at the time interval where neuroimaging was performed.

### Cerebral Energy Dysfunction in the Early Phase following SAH Is Non-Ischemic

Despite assessment of cerebral perfusion, oxygenation and hemodynamics showed absence of abnormalities, sampling of patient brain extracellular fluid obtained at the time interval of PCT revealed levels of LPR, glutamate, and glycerol that were all pathologically elevated (see Table 2). These neurometabolic abnormalities were all clinically relevant: in particular, brain LPR was on average above 40, a threshold that is associated with worse neurological recovery (17, 23, 26).

When focusing on episodes of cerebral energy dysfunction, we found that these were frequent, corresponding to two thirds of total CMD samples collected during PCTs. We found that cerebral energy dysfunction was not associated with ischemia/oligemia, but rather with normal to supranormal CBF. Previous clinical investigation in patients with traumatic brain injury revealed that energy dysfunction is predominantly non-ischemic (16, 17) and unrelated to reduced cerebral perfusion (27, 28). To the best of our knowledge, this is the first study to show evidence of non-ischemic/hypoxic cerebral energy dysfunction during the early brain injury phase following SAH in humans.

Interestingly, when dichotomizing global CBF data as normal vs. hyperemic (Table 3), hyperemia correlated with a greater impairment of cerebral energy metabolism and higher levels of cerebral glycerol, a marker of brain cell membrane disruption. Brain glutamate, a marker of brain excitotoxicity, was also higher in hyperemic conditions. In line with this, we observed that brain LPR and glycerol were positively correlated with increased CBF (Figure 2). Altogether, these findings establish a link between hyperemia and the severity of neurochemical derangements.

### Potential Mechanisms Underlying Cerebral Energy Dysfunction

Our data support the notion that mechanisms alternative to ischemia or hypoxia may be implicated in the pathogenesis of cerebral energy dysfunction following SAH. Hyperglycolysis (10, 14, 15, 29, 30), mitochondrial dysfunction (31), electrographic seizures (18), and cortical spreading depression (32) have been associated with cerebral energy dysfunction and metabolic crisis. Cerebral energy dysfunction is now well recognized as an important determinant of secondary brain injury (11) and patient prognosis (19, 33), at least in patients with traumatic brain injury.

The association between hyperemia and cerebral energy dysfunction during the early brain injury phase of SAH is not unprecedented and may help understanding underlying mechanisms. A growing body of evidence suggests that cortical spreading depression might play an important role in the pathophysiology of secondary cerebral damage following acute brain injury in general and are implicated in the early brain injury phase following SAH (34). Sakowitz and colleagues indeed found a relationship between cortical spreading depression and cerebral energy dysfunction in patients with SAH (35). Persistent impairment of cerebral energy metabolism might occur despite apparent hyperemia (36). Further study is needed to examine the exact relationship of causality between spreading depressions and cerebral energy dysfunction following SAH: it is also conceivable that treatment of spreading depressions may improve brain energetics.

Mitochondrial dysfunction is characterized by increased LPR, because of elevated brain lactate but with normal-to-elevated levels of pyruvate and PbtO_2_ (31). This cerebral metabolic pattern is distinct from that seen in ischemia where LPR elevation is coupled with low pyruvate and PbtO_2_ (23). Here, episodes of cerebral energy dysfunction were associated with normal PbtO_2_ and CBF, and also with normal-to-elevated brain extracellular pyruvate (on average 141.0 ± 99.2 µmol/L), thereby suggesting that mitochondrial dysfunction may be potentially implicated. Finally, elevated lactate may be secondary to increased activation of astrocytic glycolysis (29, 30).

### Potential Clinical Implications

Diagnosis of cerebral energy dysfunction requires advanced brain monitoring with the use of the cerebral microdialysis technique, thereby reinforcing the utility of this monitoring modality for the care of patients with poor-grade SAH (37). The fact that cerebral energy dysfunction might occur despite brain physiology (ICP and PbtO_2_) and CBF are normal (or even supranormal) indicates that therapy targeted solely to improve cerebral oxygenation and perfusion may not be enough to mitigate brain damage during the acute phase following SAH. While ischemia/hypoxia undoubtedly might play a role in the pathogenesis of SAH, the scientific paradigm has increasingly turned its interest toward the search for alternative mechanisms (7), particularly at the early brain injury phase (1, 4). Emerging evidence suggests that therapies aimed at modulating neuroenergetics might be protective after brain injury (38). Recent clinical investigation further demonstrates that resuscitation of the injured human brain with hypertonic lactate solutions exerts a beneficial effect on cerebral energy metabolism (39, 40).

### Study Limitations

The first limitation is related to the single-center observational cohort and the relatively small sample-size, which limits data generalizability. PCTs, however, were analyzed blinded to intracranial monitoring data; similarly, CMD and other brain physiological data were matched to PCTs in a blinded fashion. Although a correlation between CBF and CMD LPR and glycerol was found during episodes of cerebral energy dysfunction, due to the limited number of patients we could not use multiple regression with adjustment of cofactors, therefore we cannot confirm a causal relationship.

Timing of brain imaging is an important issue. Although brain PCT was performed at a relatively early phase after SAH (median 41 h from ictus onset) the role of initial global ischemia after SAH cannot be extrapolated from our data. Previous studies performed at the very early phase (<12 h) indeed reported acute cerebral hypoperfusion in subjects with SAH undergoing Xenon contrast-enhanced CT scanning (41). Our findings therefore apply more to the post-resuscitation ICU phase (approximately day 1–3).

Based on our local practice in patients with high grade SAH, CPP was kept >70 mm Hg (average CPP was 77 ± 9 mm Hg). As the major finding of this study was that cerebral energy dysfunction was frequent and was associated with normal to hyperemic CBF, it is unclear whether this was due only to the intrinsic SAH pathophysiology or whether this was, at least in part, caused or even exacerbated by elevated CPP. Based on these data, a preventive strategy of CPP augmentation may not seem justified in patients with SAH, since it may potentially aggravate cerebral energy dysfunction. Our data appear in line with previous microdialysis studies in patients with traumatic brain injury, showing that lower CPP (50–60 mm Hg) may be sufficient to preserve normal cerebral energy metabolism (42).

PCTs were performed in stable conditions, after initial resuscitation. This is an advantage in terms of the homogeneity of the data analyzed. However, our findings only apply to early post-SAH phase when ICP and CPP are stable, but we cannot extrapolate our findings to conditions of more disturbed intracranial physiology (e.g., intracranial hypertension). While matching of PCT with CMD and PbtO_2_ data offers a comprehensive view of the injured brain state, on the other hand it only provides a partial view of the overall picture. This is mainly because PCTs is not available continuously. Our study should therefore be considered as hypothesis generating, and further investigation is needed to confirm our findings. We believe, however, that, notwithstanding these limitations, this clinical study identifies cerebral energy dysfunction as a novel pathogenic determinant of early brain injury following SAH.

## Conclusion

Our study in patients with comatose aneurysmal SAH who underwent advanced brain monitoring with cerebral microdialysis and PbtO_2_, in combination with PCT performed during the early phase following ictus onset, indicates that cerebral energy dysfunction is frequent and is associated with normal to hyperemic CBF. Our data identify an alternative pathogenic mechanism of early brain injury following SAH in humans, which appears unrelated to cerebral ischemia/hypoxia. Management targeted to improve energy substrate delivery—together with adequate cerebral perfusion and oxygenation—may help attenuating secondary brain damage at the early phase of SAH. Additional study is needed to characterize the exact mechanisms of SAH-related cerebral energy dysfunction and to examine whether timely diagnosis and treatment may translate into better patient outcome.

## Ethics Statement

Approval for the study was obtained from the Ethical Committee of the University of Lausanne. A waiver of consent was given due to the retrospective observational cohort study.

## Author Contributions

LC collected the data, performed data analysis, and drafted the manuscript; CP and DS helped with data collection and revised the manuscript; RD, PE, and RM revised the manuscript and provided important intellectual contribution; MO conceived the study design, supervised data collection and analysis, and revised the manuscript. All the authors have read and approved the content of the present manuscript.

## Conflict of Interest Statement

The authors declare that the research was conducted in the absence of any commercial or financial relationships that could be construed as a potential conflict of interest.
